# The feasibility to use artificial intelligence to aid detecting focal liver lesions in real-time ultrasound: a preliminary study based on videos

**DOI:** 10.1038/s41598-022-11506-z

**Published:** 2022-05-11

**Authors:** Thodsawit Tiyarattanachai, Terapap Apiparakoon, Sanparith Marukatat, Sasima Sukcharoen, Sirinda Yimsawad, Oracha Chaichuen, Siwat Bhumiwat, Natthaporn Tanpowpong, Nutcha Pinjaroen, Rungsun Rerknimitr, Roongruedee Chaiteerakij

**Affiliations:** 1 Faculty of Medicine, Chulalongkorn University, Bangkok, Thailand; 2 Center of Excellence for Innovation and Endoscopy in Gastrointestinal Oncology, Division of Gastroenterology, Department of Medicine, Faculty of Medicine, Chulalongkorn University, Bangkok, Thailand; 3 Image Processing and Understanding Team, Artificial Intelligence Research Group, National Electronics and Computer Technology Center, Pathum Thani, Thailand; 4 Division of Gastroenterology, Department of Medicine, King Chulalongkorn Memorial Hospital, The Thai Red Cross Society, Bangkok, Thailand; 5 Division of Gastroenterology, Department of Medicine, Faculty of Medicine, Chulalongkorn University, Bangkok, Thailand; 6 Department of Radiology, Faculty of Medicine, Chulalongkorn University, Bangkok, Thailand; 7 Department of Radiology, Faculty of Medicine, Chulalongkorn University and King Chulalongkorn Memorial Hospital, Bangkok, Thailand

**Keywords:** Hepatocellular carcinoma, Ultrasonography, Biomedical engineering

## Abstract

Despite the wide availability of ultrasound machines for hepatocellular carcinoma surveillance, an inadequate number of expert radiologists performing ultrasounds in remote areas remains a primary barrier for surveillance. We demonstrated feasibility of artificial intelligence (AI) to aid in the detection of focal liver lesions (FLLs) during ultrasound. An AI system for FLL detection in ultrasound videos was developed. Data in this study were prospectively collected at a university hospital. We applied a two-step training strategy for developing the AI system by using a large collection of ultrasound snapshot images and frames from full-length ultrasound videos. Detection performance of the AI system was evaluated and then compared to detection performance by 25 physicians including 16 non-radiologist physicians and 9 radiologists. Our dataset contained 446 videos (273 videos with 387 FLLs and 173 videos without FLLs) from 334 patients. The videos yielded 172,035 frames with FLLs and 1,427,595 frames without FLLs for training on the AI system. The AI system achieved an overall detection rate of 89.8% (95%CI: 84.5–95.0) which was significantly higher than that achieved by non-radiologist physicians (29.1%, 95%CI: 21.2–37.0, *p* < 0.001) and radiologists (70.9%, 95%CI: 63.0–78.8, *p* < 0.001). Median false positive detection rate by the AI system was 0.7% (IQR: 1.3%). AI system operation speed reached 30–34 frames per second, showing real-time feasibility. A further study to demonstrate whether the AI system can assist operators during ultrasound examinations is warranted.

## Introduction

Ultrasound is an imaging modality of choice for screening and surveillance for hepatocellular carcinoma (HCC). It is a non-invasive procedure and machines are widely available in all levels of hospitals ranging from primary care to secondary and tertiary care. The effectiveness of HCC screening and surveillance is mainly impacted by the quality of the ultrasound examination, which depends on several factors including machine model, patient factors (e.g., body habitus, background liver parenchyma), and, most importantly, experience of ultrasound operators. Sensitivities for detection of focal liver lesions (FLLs) vary across centers in line with the experience level of the operators. A recent meta-analysis reported that ultrasound has variable sensitivities of 27.9% to 100% for detection of any-stage HCC and a range of 21.4% to 88.9% for detection of early HCC^1^. Despite the wide accessibility to ultrasound machines, the number of experienced ultrasound operators are insufficient, particularly at remote healthcare facilities^2^. To address this issue, training non-radiologist physicians or health care personnel to perform ultrasound for screening or diagnosis of specific diseases/conditions has been proposed^3–5^. Although this approach may improve HCC surveillance coverage, accuracy of FLL detection may suffer during the initial stage of the learning curve of non-radiologists and may never reach the level achieved by experienced radiologists. Developing an artificial intelligence (AI) system may help non-radiologists improve accuracy in detecting more FLLs while performing ultrasound and lead to more timely investigation and management.

Several offline AI systems for characterization of FLLs in ultrasound images have been developed and have demonstrated promising potential as a tool to support decision making for further management^6–9^. Before lesion characterization can begin, the first crucial step of any system is the ability to detect FLLs. There are some major differences between AI systems for characterization and detection of FLL. Most AI systems for characterization receive input from the FLL as a user-defined region of interest (ROI) and characterize the FLL in an offline manner^8,9^. In contrast, the AI system for detection must find FLLs in the vast liver background which is comprised of other interfering non-lesion organ structures and artifacts. Considerable noise is present during ultrasound acquisition, detecting FLLs is therefore quite challenging. The non-lesion structures seen in ultrasound may appear in various configurations and often resemble FLLs. For example, cross-sectional blood vessels can mimic cysts. In order to deliver full clinical utility, AI systems must operate in a real-time manner by detecting FLLs while ultrasound is being performed.

In this study, we demonstrated the feasibility of AI to aid detecting FLLs during ultrasound. An AI system for FLL detection in ultrasound videos was developed as part of this study. The AI system was trained with a two-step process using large datasets of ultrasound snapshot images and video frames. We further compared the detection performance of the AI system to performance achieved by non-radiologist physicians and radiologists to demonstrate its feasibility to improve FLL detection rates.

## Methods

The video data were prospectively collected at a university hospital. The study was compliant to the Health Insurance Portability and Accountability Act (HIPAA) and approved by the Institutional Review Board of Chulalongkorn University, Bangkok, Thailand (IRB No. 533/63). Informed consent was obtained from each patient before recording the ultrasound videos. All data were de-identified and analyzed anonymously. The study protocol strictly adhered to the ethical guidelines of the Declaration of Helsinki.

### Dataset

We prospectively enrolled patients who visited the Division of Gastroenterology, King Chulalongkorn Memorial Hospital, Bangkok, Thailand, for HCC surveillance between January 2019 and December 2020. Full-length ultrasound videos with the frame rate of 30 frames per second (FPS) were collected at the time of ultrasound examination. We collected full-length videos of the entire ultrasound examination which more accurately represented an ultrasound examination in clinical settings. Since FLLs usually only appeared briefly during the whole ultrasound examination, evaluation of the AI performance on the full-length videos was more likely to mirror performance when used in clinical practice.

**Figure 1:**
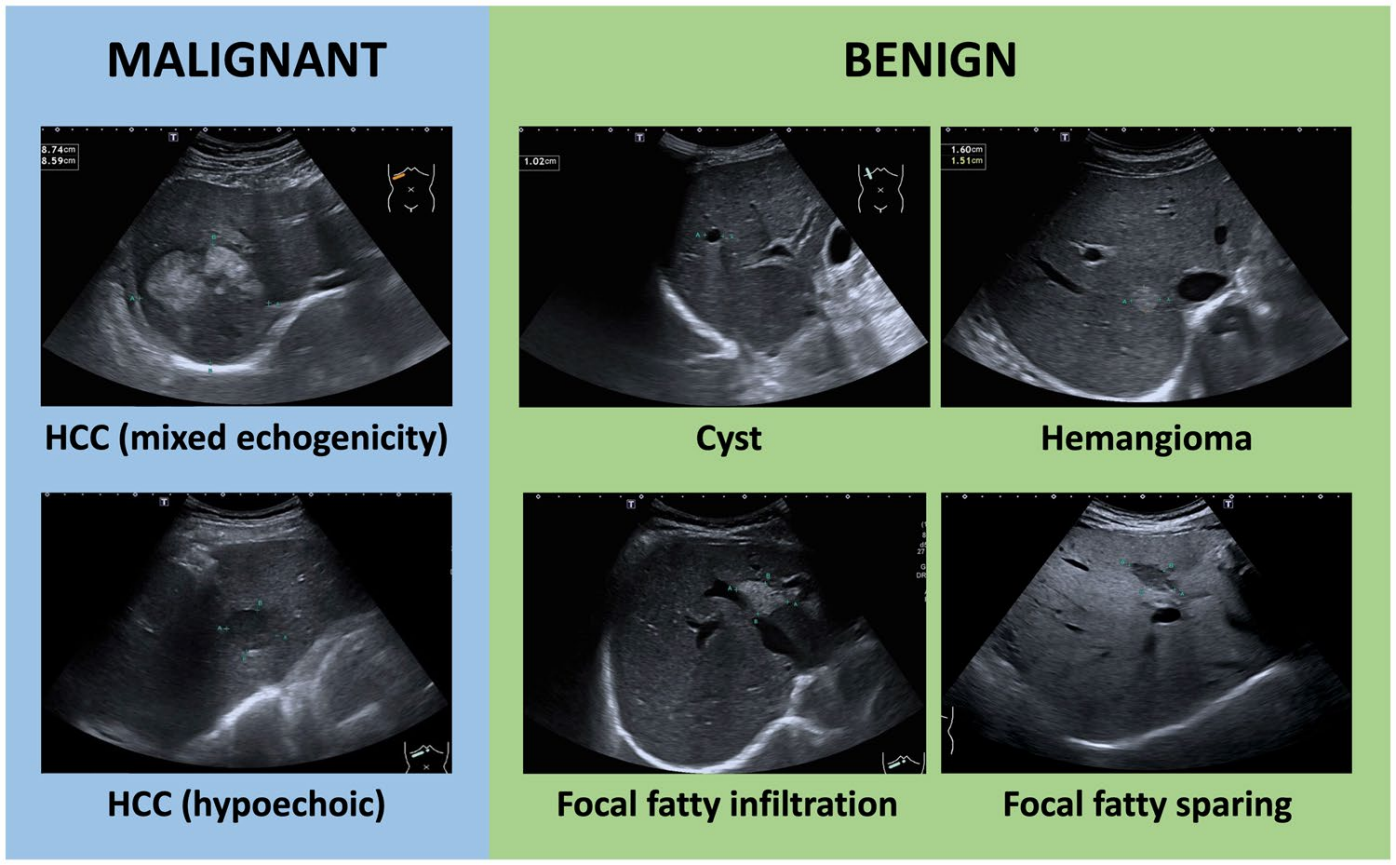
Examples of focal liver lesions. The lesions are indicated with blue markers.

**Figure 2:**
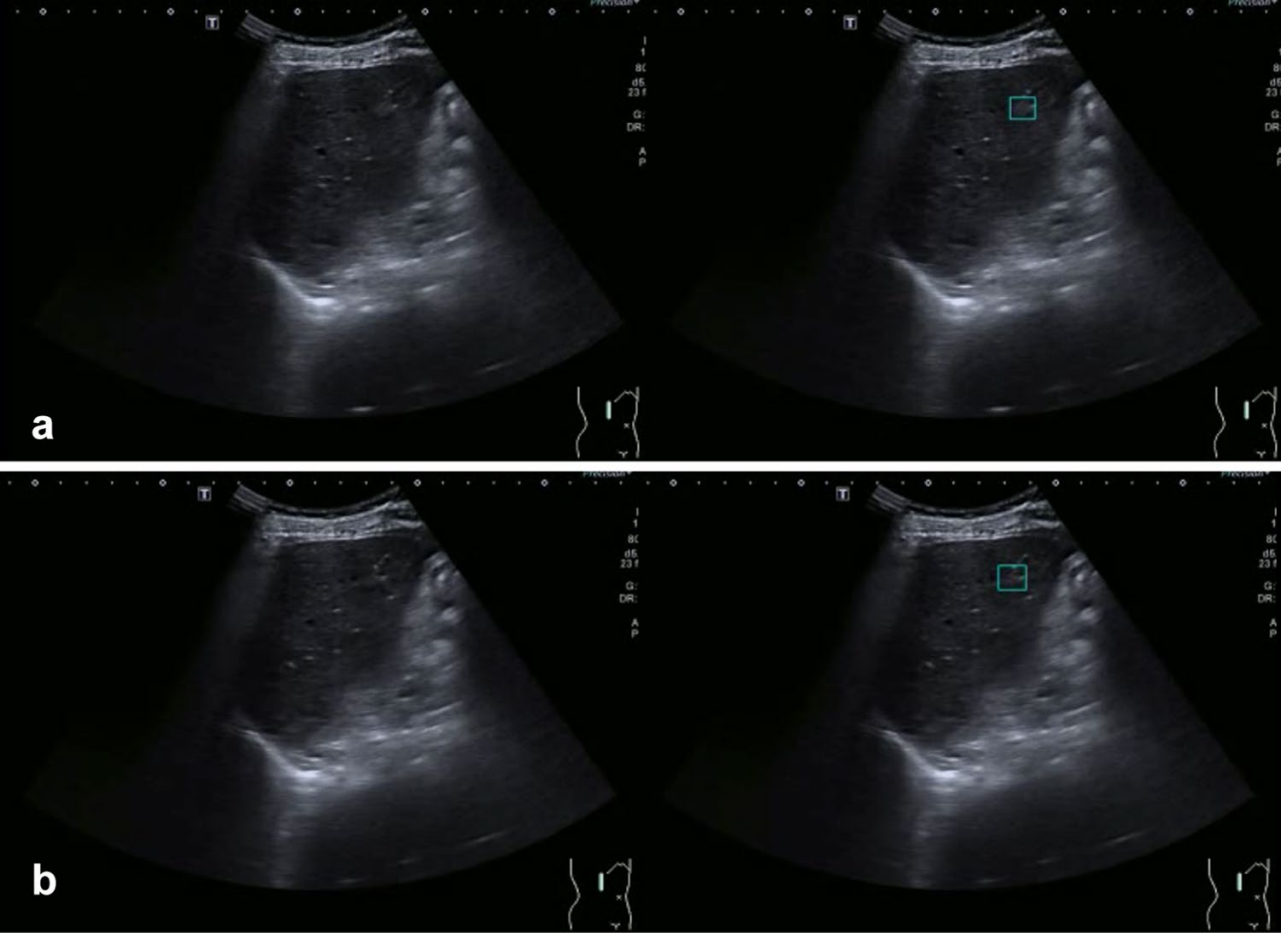
Example of the same lesion appearing as a distinct observation when the ultrasound beam passes through the center of the lesion (2**a**). In contrast, the lesion appears as a small faint observation when the ultrasound beam passes through the periphery of the lesion (2**b**). Left panels show the original frames. Right panels show the labeled location of the lesion.

Five of the most commonly encountered FLLs including HCCs, cysts, hemangiomas, focal fatty sparings (FFSs) and focal fatty infiltrations (FFIs) were the selected targets for this study (Fig. 1)^10,11^. Although FFS is not considered as a true FLL, it was included because it is a common finding in liver ultrasound examinations and needs to be differentiated from true focal liver lesions. The definitive diagnoses of FLLs were verified using pathology or typical characteristics on magnetic resonance imaging or computerized tomography images. HCCs previously treated by locoregional modalities were excluded. FLLs in all videos were manually labeled for diagnosis and location by bounding box using *DarkLabel* open-source software (https://github.com/darkpgmr/DarkLabel). Since each lesion appeared in multiple video frames, we labeled all frames with appearing lesions including frames in which the lesion appeared distinctively (Fig. 2a) and frames in which the lesion appeared faintly (Fig. 2b). Using data labeled by this method for training enabled the AI system to detect lesions during rapid probe movement when ultrasound is performed in practice. We also included videos without lesions to train the AI system to differentiate lesions from liver parenchyma and non-lesion structures.

The video dataset was randomly divided into training, tuning and test sets. The training set was used to train the AI system. The tuning set monitored performance during the training process. The test set was used for final evaluation of the AI performance. The dataset was randomly split at the patient level with videos from the same patient allocated to the same set, ensuring all sets were completely independent. Furthermore, we included only 1 video per patient in the test set to minimize intra-patient correlation in performance evaluation.

### AI system development

**Figure 3:**
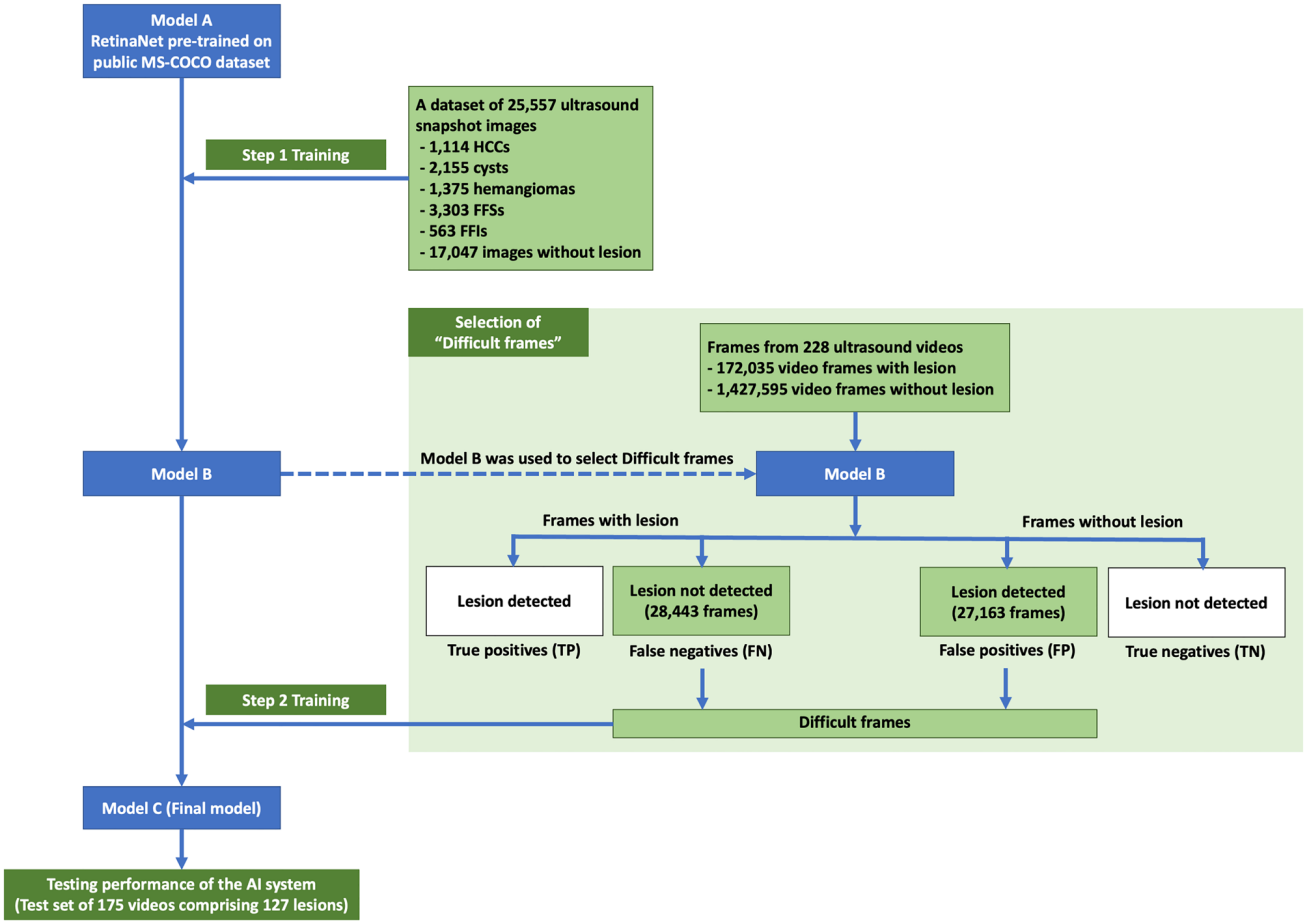
AI system development.

We trained the RetinaNet model^12^, which is an architecture based on convolutional neural networks (CNN), by a supervised learning method. We used a two-step training strategy (Fig. 3), as follows:

#### Step 1 training

The purpose of this step was to pre-train the AI system. First, we adopted the RetinaNet model implemented in Keras (https://github.com/fizyr/keras-retinanet) ^12–14^. A hallmark of RetinaNet is Focal Loss, which facilitates training of the model to detect a limited number of object(s) within a vast area of background^12^, as was our case where only 1 or a few FLL(s) appeared in the ultrasound probe’s field of view. Being a one-stage detector, RetinaNet has a good balance between detection performance and inference speed, which is essential for real-time detection. The RetinaNet contains a large number of parameters that should be pre-trained on a large image dataset before fine-tuning on our specific image dataset. Several studies have shown that accurate object detectors should be pre-trained on a fully-annotated image corpus such as MS-COCO^15^ that comprises 330,000 fully-annotated images with 1.5 million object instances. *Model A* corresponded to the RetinaNet pre-trained on the MS-COCO dataset. We then further trained the *Model A* with a subset of our previously collected dataset^7^ of 8510 ultrasound snapshot images of FLLs comprising 1114 HCCs, 2155 cysts, 1375 hemangiomas, 3303 FFSs, 563 FFIs and 17,047 snapshot images without FLL, resulting in *Model B*. Our previous study^7^ used ultrasound snapshot images to train a RetinaNet model to detect FLLs. In Step 1 of the current study, we trained the *Model A* with the same training hyperparameters (Supplementary Method S1). Although the dataset of snapshot images contained a large number of FLLs with various characteristics, we acknowledged that the ultrasound snapshot images were intentionally captured at clear standard views or captured when an FLL was most clearly visible. However, this is not the case during real-time ultrasound acquisition. To enable the model to detect FLLs when not clearly seen, we further trained the model using video frames. In the ultrasound videos, FLLs were not always clearly visible, and there were an extensive number of video frames containing non-lesion structures and artifacts.

#### Selection of difficult frames

We found that *Model B* which was trained by ultrasound snapshot images captured at clear standard views alone could not always detect FLLs in some video frames in which lesions appeared as faint observations. It also occasionally produced false positives for non-lesion structures in video frames. We labeled this grouping of video frames as ‘*difficult frames*’. We selected the difficult frames in the videos in the training set by using *Model B* to predict outputs on all video frames. The resulting outputs were grouped as true positives (TP), true negatives (TN), false positives (FP) and false negatives (FN) as in Fig. 3. Video frames in the Group FN and FP were counted as the difficult frames.

**Figure 4:**
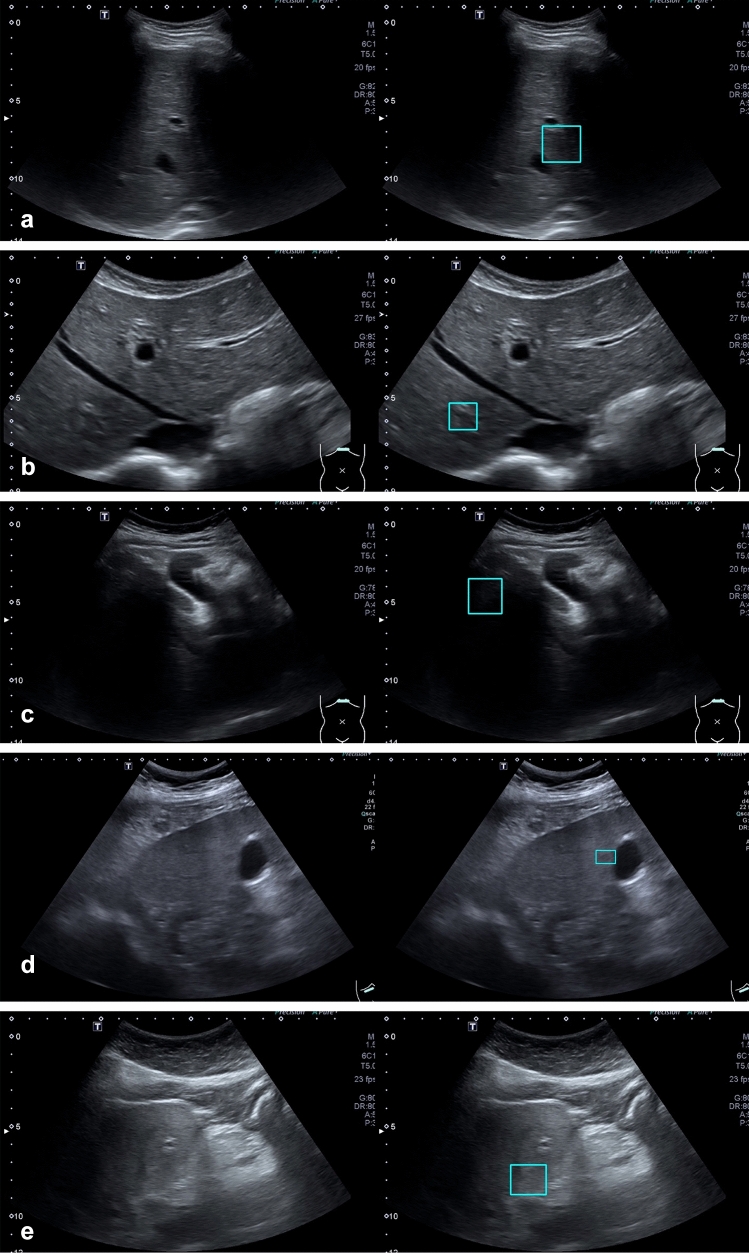
Difficult frames in Group FN used to train the AI system in the Step 2 Training. These are frames with faint lesions not detected by Model B pre-trained on ultrasound snapshot images in the Step 1 Training. Left panels show the original frames. Right panels show labeled location of the lesions (**a**: HCC, **b**: cyst, **c**: hemangioma, **d**: FFS, **e**: FFI).

Most of the difficult frames in Group FN were frames in which lesions appeared faintly (Fig. 4). Our full-length videos contained frames in which lesions appear as either distinct or faint observations in contrast to snapshots where the lesions are intentionally taken at well-visualized angles. Using frames in the Group FN for further training enabled the AI system to detect faint-appearing lesions which would happen when the ultrasound probe was not placed at the center of the lesion or was moving rapidly, when the lesion was partially obscured by shadows, or when there was poor ultrasound beam penetration.

**Figure 5:**
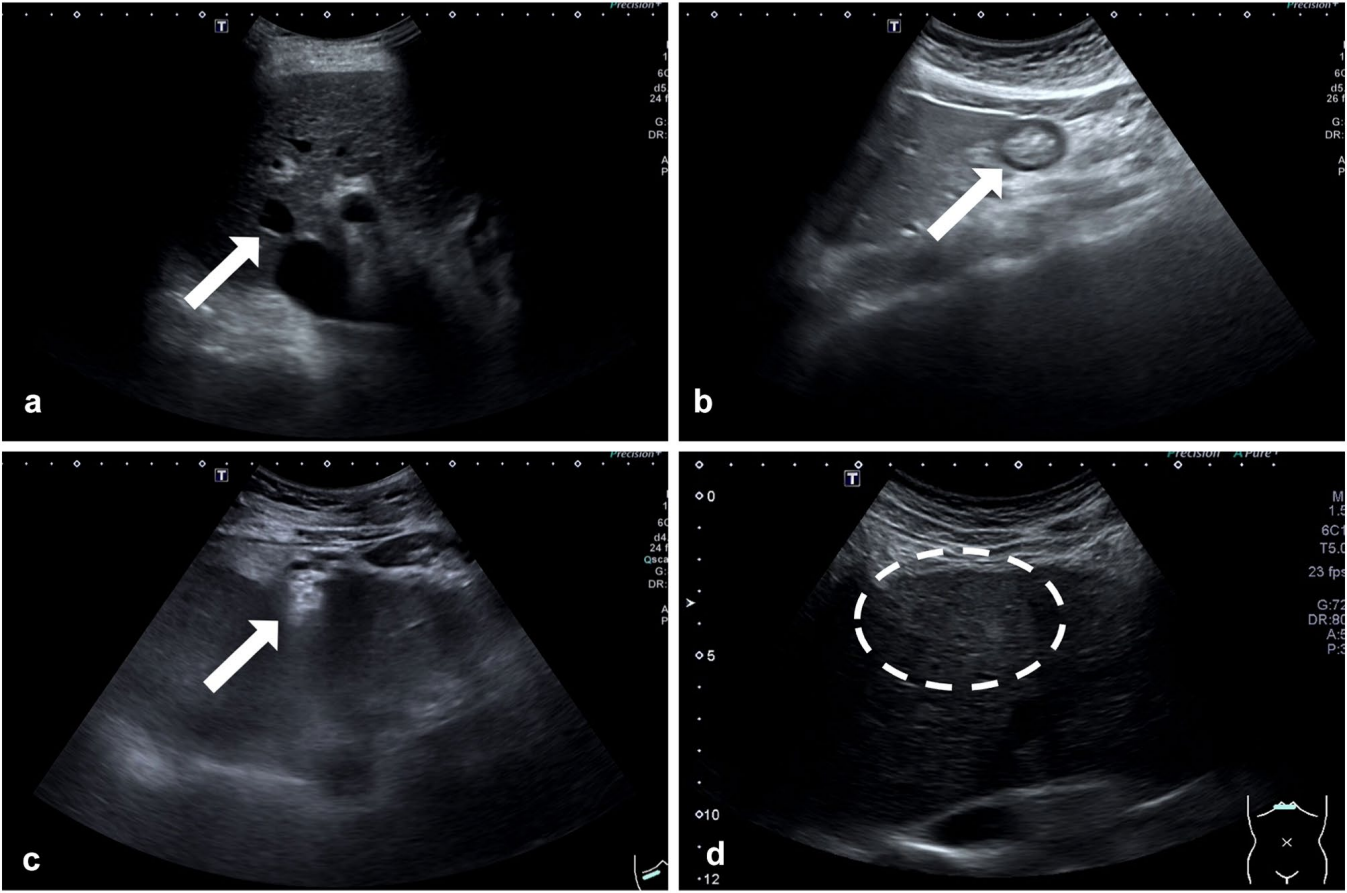
Difficult frames in Group FP used to train the AI system in the Step 2 Training. These are frames in which Model B pre-trained on ultrasound snapshot images in the Step 1 Training falsely detected other structures (arrows) as FLLs (**a**: blood vessel, **b**: stomach, **c**: fibrous tissue, **d**: parenchymal heterogeneity (dashed circle)).

Video frames in Group FP contained various non-lesion structures, other organs, atypical views of liver and artifacts which were not routinely captured as image snapshots in the picture archiving and communication system (PACS) in radiology, but commonly encountered during real-time ultrasound (Fig. 5). Using video frames in Group FP for training enabled the AI system to more accurately ignore areas without lesions, thus minimizing the false positive detection of FLLs.

#### Step 2 training

We trained *Model B* with the *difficult frames* (i.e., Group FN and FP) to yield *Model C*, which was our final AI system. Since each video often contained similar frames, using all frames to train the AI system would cause the AI system to overfit to these scenes. Examples of scenes with limited frame variability include events when the operator freezes the screen or when the ultrasound probe is placed still for an extended time. To maintain high variance relative to the number of frames used for training, we selected only significantly different frames from the pool of difficult frames to train the AI system. We calculated dissimilarity between frames and used only frames with dissimilarity values greater than a selected threshold for training (Supplementary Method S2). The training hyperparameters of the AI system are described in Supplementary Method S1.

#### Tuning the AI system during training process

The tuning set was used to monitor performance of the AI system during the training process and to adjust the hyperparameters of the AI system. After every epoch of training, the AI system was evaluated on the tuning set using detection rates and false positives as evaluation metrics. Training was stopped when performance had not improved for 5 epochs. In addition, after each training experiment, hyperparameters of the AI system were adjusted to optimize performance on the tuning set.

#### Testing performance of the AI system

Finally, the AI system was evaluated on videos in the test set. To ensure that performance evaluation was completed in an unbiased manner, each video was evaluated on all frames with either distinct or faint appearing lesions as well as frames without lesion. We also applied a heuristic method to reduce false positive prediction. False positive detections often appeared as a flicker in only 1 frame. In contrast, if there was a true lesion, the AI system outputted bounding boxes in contiguous frames. The heuristic method allowed the AI system to predict that there was a lesion only when the lesion was detected in at least 2 consecutive frames.

### Evaluation metrics for the AI system

We evaluated the AI performance by the following metrics (details in Supplementary Method S3).

#### Per-lesion detection rate

This was the primary outcome in our study. It was defined using the following formula:

$$detection\;rate = \frac{number\;of\;detected\;FLLs}{{number\; of\;all\;FLLs}}$$

We also calculated detection rate stratified by FLL diagnosis. For example, HCC detection rate was defined using the following formula:

$$HCC\;detection\;rate = \frac{number\; of\;detected\;HCCs}{{number\;of\;all\;HCCs}}$$

#### False positive detection rate

When the AI system falsely detected other organ structures as FLLs, these frames were counted as false positives. We calculated false positive detection rate for each video by the following formula:

$$false\;positive\;detection\;rate = \frac{number\;of\;false\;positive \;frames}{{number\;of\;all\;frames\;in\;the\;video}}$$

We then reported the median and interquartile range (IQR) of false positive detection rates, aggregated across all videos in the test set.

### Comparison of FLL detection rates between the AI system and physicians

Performance of the AI system in the true positive detection of FLLs was compared to the performance of 25 physicians including 16 non-radiologist physicians who are considered non-experts (years of practice: mean 4.6, SD 1.2, range 1–6) and 9 radiologists who are considered experts (years of radiology practice: mean 3.9, SD 1.1, range 3–5; number of liver ultrasound exams previously performed: mean 1009, SD 640, range 180–2000). The full-length videos in the test set were randomly assigned to the physicians. The videos reviewed by each group of physicians constituted the whole test set of 175 videos with individual non-radiologist physician and radiologist reviewing 9–12 and 18–20 videos, respectively. The physicians were blinded to patient medical records and independently reviewed the videos without time constraint or attempt limits. The video review process was done in *VIA Annotation Software*^16^. This software allows physicians to either view a video as a continuous cine or frame by frame. Upon seeing an FLL, a physician drew a bounding box around it. If the bounding box overlapped with ground truth label of a lesion in at least 1 frame, the lesion was counted as detected by the physician. In contrast, if the bounding box drawn by a physician did not overlap with any ground truth label, it was counted as a false positive. Sum of the false positives across all videos was reported.

**Figure 6:**
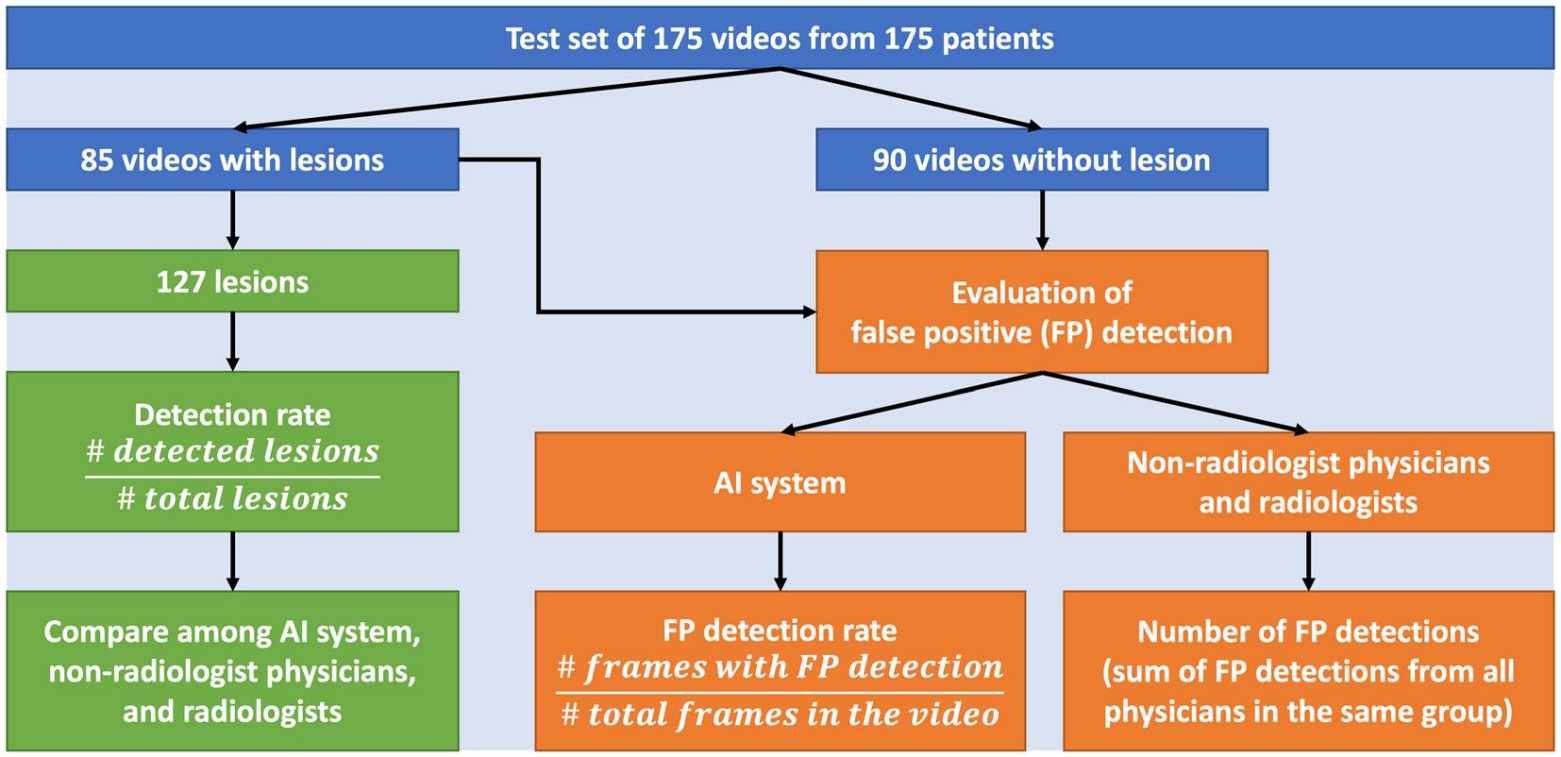
Process in performance evaluation of the AI system, non-radiologist physicians and radiologists. Detection rates by the AI system and physicians were evaluated using the same formula. Evaluation of false positive (FP) detection were different for the AI system and physicians, thus they should not be directly compared. Specifically, the AI system was evaluated by false positive detection rate, while physicians were evaluated by number of false positive detections.

The false positivity of the AI system and physicians were not defined in the same way. For example, a single structure mimicking FLL can appear in contiguous frames. Physicians would draw only 1 bounding box on 1 frame of the false positive structure, and this would be counted as one false positive. In contrast, the AI system might repeatedly output bounding boxes on multiple frames having the false positive structure and all bounding boxes were counted towards false positive detection rate. Accordingly, we did not compare the false positivity of the AI system and physicians. Only detection rates can be fairly compared between the physicians and the AI system. The process of the performance evaluation of the AI system and physicians is summarized in Fig. 6.

### Statistical analysis

Python version 3.7 (Python Software Foundation, Delaware, USA) was used for development of the AI system and formal analyses. Performance results of the AI system were reported as an overall detection rate, detection rates stratified by FLL diagnosis, and false positive detection rate. Performance results of the physicians were reported as detection rates and number of false positive detections. All rates are presented with corresponding 95% confidence intervals (CI). Detection rates of the AI system were then compared to rates from the non-radiologists and radiologists using McNemar’s test implemented in Stata Statistical Software version 15.1 (StataCorp LLC, College Station, TX, US). The two-tailed significance level (ɑ) was set at 0.05.

### Ethics statement

The study was compliant to the Health Insurance Portability and Accountability Act (HIPAA) and approved by the Institutional Review Board of Chulalongkorn University, Bangkok, Thailand (IRB No. 533/63). Informed consent was obtained from each patient before data collection. All data were de-identified and analyzed anonymously. The study protocol strictly adheres to the ethical guidelines of the Declaration of Helsinki.

## Results

### Dataset characteristics

Table 1 presents characteristics of video datasets including 446 videos from 334 patients. There were 273 videos with 387 FLLs (some videos contained more than 1 lesion) and 173 videos without FLLs. The diagnoses of the 387 FLLs were 71 (18.3%) HCCs, 138 (35.7%) cysts, 78 (20.2%) hemangiomas, 69 (17.8%) FFSs and 31 (8.0%) FFIs. The median (interquartile range, IQR) sizes of HCC, cyst, hemangioma, FFS and FFI were 2.2 (2.0), 1.1 (0.4), 1.4 (0.9), 1.2 (0.6) and 1.9 (0.9) cm, respectively.

**Table 1:** Dataset characteristics.

Number	All sets	Training set	Tuning set	Test set
Patients	334	123	36	175
Total videos	446	228	43	175
Videos with lesions	273	145	43	85
Types of lesions				
Total	387	199	61	127
HCC	71	39	9	23
Cyst	138	84	20	34
Hemangioma	78	38	13	27
FFS	69	24	15	30
FFI	31	14	4	13
Videos without lesions	173	83	0	90
Frames with lesions	267,820	172,035	40,923	54,862
Frames without lesions	1,879,727	1,427,595	162,338	289,794
Difficult frames with lesions (Group FN)	NA	28,443	NA	NA
Difficult frames without lesions (Group FP)	NA	27,163	NA	NA
Median size in cm (IQR)				
HCC	2.2 (2.0)	2.3 (3.1)	2.1 (1.8)	1.7 (1.0)
Cyst	1.1 (0.4)	1.0 (0.4)	1.4 (0.5)	1.3 (0.5)
Hemangioma	1.4 (0.9)	1.5 (0.8)	1.5 (0.5)	1.2 (0.9)
FFS	1.2 (0.6)	1.2 (0.6)	1.1 (1.3)	1.1 (0.4)
FFI	1.9 (0.9)	2.0 (0.4)	1.9 (0.7)	1.8 (0.9)

*NA* not applicable; *HCC* hepatocellular carcinoma; *FFS* focal fatty sparing; *FFI* focal fatty infiltration; *FN* false negative; *FP* false positive; *IQR* interquartile range.

The training set included 228 videos (145 videos with FLLs and 83 videos without FLLs), comprising 172,035 frames with FLLs and 1,427,595 frames without FLLs. After selection of difficult frames, 28,443 difficult frames with FLLs (Group FN) and 27,163 difficult frames without FLLs (Group FP) were used to train the AI system in the *Step 2 Training*.

In the test set of 175 videos, 85 videos contained 127 FLLs including 23 (18.1%) HCCs, 34 (26.8%) cysts, 27 (21.3%) hemangiomas, 30 (23.6%) FFSs and 13 (10.2%) FFIs, and 90 videos did not have FLLs. The median (IQR) sizes of HCC, cyst, hemangioma, FFS and FFI in the test set were 1.7 (1.0), 1.3 (0.5), 1.2 (0.9), 1.1 (0.4) and 1.8 (0.9) cm, respectively. Supplementary Table S1 describes characteristics of FLLs in the test set. There were FLLs with various sizes and echogenicity patterns in the test set. Of the 23 HCCs included in the test set, 15 (65.2%) were early HCCs (size < 2 cm). HCCs with hypoechogenicity, hyperechogenicity and heterogeneous echogenicity patterns were all included. For hemangiomas, both typical hyperechoic lesions (21/27, 77.8%) and atypical hypoechoic/heterogeneous lesions (6/27, 22.2%) were included.

### Performance of AI system

Performance of the AI system on the test set are summarized in Table 2. The AI system achieved an overall detection rate of 89.8% (95%CI: 84.5–95.0) (114/127 lesions). When stratified by each FLL diagnosis, the lesion detection rates were 100% (95%CI: 85.2–100) for HCCs (23/23), 82.4% (95%CI: 69.5–95.2) for cysts (28/34), 85.2% (95%CI: 71.8–98.6) for hemangiomas (23/27), 96.7% (95%CI: 90.2–100) for FFSs (29/30) and 84.6% (95%CI: 65.0–100) for FFIs (11/13). The AI system did not detect 13/127 FLLs. Examples of undetected FLLs are shown in Supplementary Fig. S1. Median false positive detection rate was 0.7% (IQR: 1.3%).

**Table 2:** Performance results of AI system, non-radiologist physicians and radiologists on the test set.

	AI system	Non-radiologist physicians	*p* value (AI vs. non-radiologist physicians)	Radiologists	*p* value (AI vs. radiologists)
Per-lesion detection rate					
Overall	89.8% (84.5–95.0)	29.1% (21.2–37.0)	< 0.001	70.9% (63.0–78.8)	< 0.001
HCC	100% (85.2–100)	39.1% (19.2–59.1)	< 0.001	69.6% (50.8–88.4)	0.016
Cyst	82.4% (69.5–95.2)	29.4% (14.1–44.7)	< 0.001	73.5% (58.7–88.4)	0.38
Hemangioma	85.2% (71.8–98.6)	40.7% (22.2–59.3)	< 0.001	77.8% (62.1–93.5)	0.73
FFS	96.7% (90.2–100)	13.3% (1.2–25.5)	< 0.001	63.3% (46.1–80.6)	0.006
FFI	84.6% (65.0–100)	23.1% (0.2–46.0)	0.008	69.2% (44.1–94.3)	0.63
Median (IQR) false positive detection rate	0.7% (IQR 1.3%)	NA	NA	NA	NA
False positive detections	NA	118 false positive detections from 175 videos	NA	204 false positive detections from 175 videos	NA

95% CIs are shown in brackets.

*NA* not applicable; *HCC* hepatocellular carcinoma; *FFS* focal fatty sparing; *FFI* focal fatty infiltration; *IQR* interquartile range.

To demonstrate feasibility of the AI system for real-time detection of FLLs during ultrasound surveillance for HCC, it must be able to operate at a frame rate of at least 25 frames per second (FPS)^17^. The developed AI system achieved a good balance between detection performance and processing time per frame. When tested on various commonly available graphics processing units (GPU), the AI system was able to operate at 30 FPS on Nvidia RTX 2080 GPU, 32 FPS on Nvidia RTX 3080 GPU and 34 FPS on Nvidia RTX 3090 GPU, showing feasibility to run in real-time. Supplementary Video S1 shows the AI system detecting an FLL in an ultrasound video.

### Comparison of detection rates between AI system and physicians

The overall detection rate of the AI system (89.8%, 95%CI: 84.5–95.0) was significantly higher than the detection rate of non-radiologist physicians (29.1%, 95%CI: 21.2–37.0, McNemar’s test ${\chi }^{2}$ = 77.0, *p* < 0.001) and radiologists (70.9%, 95%CI: 63.0–78.8, McNemar’s test ${\chi }^{2}$ = 16.0, *p* < 0.001). Comparison of detection rates stratified by definitive diagnoses between the AI system and physicians are presented in Table 2. There were 118 and 204 false positive detections in the non-radiologist and radiologist group, respectively (Table 2).

## Discussion

Our study demonstrated feasibility of AI to aid detecting FLLs during ultrasound. The AI system detected FLLs in ultrasound videos with an overall detection rate of 89.8%. It was also able to operate at 30–34 FPS on standard GPUs, showing feasibility to run in real-time. The overall FLLs detection rate of our AI system was significantly higher than both non-radiologists (29.1%) and radiologists (70.9%).

The detection rates of the AI system differed across FLL diagnoses. The AI system detected HCCs and FFSs with high detection rates of 100% and 96.7%, respectively. The detection rates were lower for hemangiomas (85.2%), FFIs (84.6%) and cysts (82.4%). Upon reviewing the videos for undetected lesions, we found that the undetected hemangiomas were of small size (< 0.5 cm) (Supplementary Fig. S1a) appearing as atypical hypoechoic lesions in the background of steatotic liver parenchyma (Supplementary Fig. S1b) and appeared faintly or obscured by shadow artifacts. We postulate that collecting more videos with atypical hemangiomas as well as videos with difficult-to-visualize lesions might ameliorate the problem. Regarding the undetected cysts, we found that our AI system sometimes misinterpreted cysts as cross-sectional blood vessels (Supplementary Figure S1c). In contrast, a cross-sectional blood vessel could also be falsely detected as a cyst, thus yielding false positive detection. A possible solution is the incorporation of a doppler mode which could identify blood vessels if flow is present. In practice, radiologists differentiate between cysts and blood vessels by using temporal information between frames such as the change in appearance when the probe is placed in different angles. However, the RetinaNet model received inputs as individual frames and detected lesions frame by frame without accounting for temporal relationships between frames. We have tried to address this issue by applying the heuristic method and found that this method might partly solve the problem. We postulate that time-sequential models might better capture the relationship between frames and better differentiate between cysts and blood vessels.

Another consideration in the evaluation of AI system performance is false positive detection. When AI systems are implemented in practice, less experienced operators may be distracted by the false positive detections. We have introduced several approaches to reduce false positive detections. First, our training data contained a wide spectrum of frames without lesions. Second, we performed a sampling technique to select difficult frames where AI might falsely detect other structures as FLLs and used them for training in the *Step 2 Training*. Lastly, we applied the heuristic method. Our AI system had a median false positive detection rate of 0.7%, which is acceptable^17–19^. We have tested the AI system in our service and found that this level of false positive detections did not cause much distraction for the user. False positive detections also did not appear in contiguous frames, which allowed users to reject these false positive detections with high confidence.

Previous studies have focused on developing AI systems for characterization of FLLs in ultrasound snapshot images^6–9^. A recent study developed an AI system for detection of FLLs in ultrasound images, which achieved an overall detection rate of 91.1%^20^. However, this reported performance cannot be directly compared to our study because they evaluated the AI system performance using ultrasound images of FLLs captured from cine loops, and images without FLLs were not included in their study. In contrast, our study evaluated the AI system performance using videos of the entire ultrasound examination. Each video included frames in which an FLL appeared as both distinct and ill-defined observations, as well as frames with non-lesion structures and artifacts.

Our study demonstrated several strengths. First, we collected full-length videos to closely represent ultrasound examinations in clinical settings. Our full-length videos contained a large and varied number of frames with and without lesions. The AI system can detect not only distinct lesions but also faint-appearing lesions which occur frequently during probe movement in an attempt to find FLLs. Second, we compared the performance of the AI system to that of physicians to determine the system’s feasibility to improve FLL detection compared to routine practice. Lastly, our AI system achieved an operation speed of 30–34 FPS, showing feasibility to run in real-time during ultrasound examination.

Our study has some limitations. First, videos in this study were collected from a single institution. We acknowledged this limitation and addressed it by using our previous large dataset of image snapshots to pre-train the AI system in the first training step. This dataset contained ultrasound images collected from multiple institutions in Thailand and had a large variety of FLL characteristics and images from 17 ultrasound machine models^7^. We believe that the pre-training process using this dataset allowed the AI system to better handle variation in ultrasound exams. Second, the AI system in this study mainly focused on FLLs detection, but not on characterization. We acknowledged that performing detection and characterization in an end-to-end process would streamline the workflow in HCC surveillance. However, the characterization system is not the focus of this current study. AI systems for differentiating benign from malignant focal liver lesions is an active area of research. Previous studies have developed AI systems for such characterization task in different imaging modalities, including computerized tomography^21–25^, magnetic resonance imaging^26,27^, contrast-enhanced ultrasound^28–30^ and B-mode ultrasound^6–9,31–33^. Lastly, this study was a proof of concept for using the AI system to detect FLLs in ultrasound videos. The results only indicated that the AI system was able to find and recognize displayable and visible FLLs. However, ultrasound examinations are operator dependent. The detection of FLLs on an ultrasound examination depends on both the ability of the operator to perform a complete scan to find FLLs and an ability to recognize FLL (given the lesion is scanned through by the operator) amid background liver parenchyma and other non-lesion structures. We believe that our AI system may potentially help improve the latter process. Nonetheless, ultrasound scans by non-expert operators may be incomplete, and thus FLLs in some area(s) of the liver may not be visualized by the operators. Accordingly, the AI system would not assist the operator to detect the unexposed FLLs. Owing to this reason, the validation in this study remained incomplete. It was only a part in the AI development workflow, which comprises both pre-clinical and clinical validation. To demonstrate the usefulness of the AI system in a clinical setting, a study using the AI system to improve clinical outcomes in the target population is needed. A clinical validation study to evaluate whether the AI system can assist operators during ultrasound examination and increase detection rates of FLLs is warranted.

## Conclusion

The AI system detects FLLs in ultrasound videos with high detection rates and acceptable false positive detection rate. It also shows feasibility to operate in real-time. To demonstrate its clinical applicability, a study using the AI system to assist operators during ultrasound examinations is needed.

## Supplementary Information

Supplementary Information 1.

Supplementary Video 1.

## Data Availability

All relevant data are within the manuscript and its Supplementary Information files.
