# Supplementary material for: The feasibility to use artificial intelligence to aid detecting focal liver lesions in real-time ultrasound: a preliminary study based on videos
Source: Sci Rep. 2022 May 11;12:7749. doi: 10.1038/s41598-022-11506-z (PMC9095624; doi:10.1038/s41598-022-11506-z)
Supplement: Supplementary file 1 — Supplementary Information 1. [file 41598_2022_11506_MOESM1_ESM.pdf]

Supplementary Information

The feasibility to use artificial intelligence to aid detecting focal liver lesions in real-time ultrasound: A preliminary study based on videos

Thodsawit Tiyarattanachai[1], Terapap Apiparakoon[2], Sanparith Marukatat[3], Sasima Sukcharoen[4], Sirinda Yimsawad[2], Oracha Chaichuen[5], Siwat Bhumiwat[6], Natthaporn Tanpowpong[7], Nutcha Pinjaroen[6], Rungsun Rerknimitr[2], Roongruedee Chaiteerakij[2]*

[1]Faculty of Medicine, Chulalongkorn University, Bangkok, Thailand
[2]Center of Excellence for Innovation and Endoscopy in Gastrointestinal Oncology, Division of Gastroenterology, Department of Medicine, Faculty of Medicine, Chulalongkorn University, Bangkok, Thailand
[3]Image Processing and Understanding Team, Artificial Intelligence Research Group, National Electronics and Computer Technology Center, Pathum Thani, Thailand
[4]Division of Gastroenterology, Department of Medicine, King Chulalongkorn Memorial Hospital, The Thai Red Cross Society, Bangkok, Thailand
[5]Division of Gastroenterology, Department of Medicine, Faculty of Medicine, Chulalongkorn University, Bangkok, Thailand
[6]Department of Radiology, Faculty of Medicine, Chulalongkorn University, Bangkok, Thailand
[7]Department of Radiology, Faculty of Medicine, Chulalongkorn University and King Chulalongkorn Memorial Hospital, Bangkok, Thailand

Table of Contents

	**Page**
**Supplementary Method S1.** Training hyperparameters of the AI system	2
**Supplementary Method S2.** Calculation of dissimilarity between frames	4
**Supplementary Method S3.** Evaluation metrics for the AI system	5
**Supplementary Table S1.** Characteristics of focal liver lesions in the test set	6
**Supplementary Figure S1.** Example lesions in the test set not detected by the final AI system	7
**Supplementary Video S1.** AI system detecting a focal liver lesion in an ultrasound video	8

Supplementary Method S1. Training hyperparameters of the AI system

Step 1: Training with large dataset of ultrasound snapshot images

**Training hyperparameters**	**Values**
Iterations (= [epochs] x [steps per epoch])	500,000
Batch size	2
Input size (width, height)	(1333, 800)
Image mean (grayscale)	46.15
Initial learning rate	0.0001
Learning rate scheduling	Decrease learning rate by a factor of 0.5 if there is no decrease in validation loss for 2 epochs
Anchor sizes	[32, 64, 128, 256, 512]
Anchor strides	[8, 16, 32, 64, 128]
Anchor ratios	[0.5, 1, 2]
Anchor scales	[1, $2^{1/3}$, $2^{2/3}$]
alpha	0.25
gamma	2.0
IoU for Negative overlap	< 0.2
IoU for Positive overlap	> 0.5
**Image augmentation**	
- Rotation	± 0.1 radian
- Translation factor	± 0.1 in x and y direction
- Scaling factor	± 0.1
- Horizontal flip probability	0.2
- Contrast range	(0.9, 1.1)
- Brightness range	(0.9, 1.1)
- Hue parameter range	(-0.05, 0.05)
- Saturation range	(0.95, 1.05)
- Motion blur	Motion in x-direction (horizontal)

Step 2: Training with difficult frames from ultrasound videos

**Training hyperparameters**	**Values**
Iterations (= [epochs] x [steps per epoch])	493,000
Batch size	4
Input size (width, height)	(576, 768)
Image mean (grayscale)	46.15
Initial learning rate	0.00001
Learning rate scheduling	Decrease learning rate by a factor of 0.5 if there is no decrease in validation loss for 2 epochs
Anchor sizes	[32, 64, 128, 256, 512]
Anchor strides	[8, 16, 32, 64, 128]
Anchor ratios	[0.5, 1, 2]
Anchor scales	$[1, 2^{1/3}, 2^{2/3}]$
alpha	0.25
gamma	1.0
IoU for Negative overlap	< 0.2
IoU for Positive overlap	> 0.5
**Image augmentation**	
- Rotation	± 0.1 radian
- Translation factor	± 0.1 in x and y direction
- Scaling factor	± 0.1
- Horizontal flip probability	0.2
- Contrast range	(0.9, 1.1)
- Brightness range	(0.9, 1.1)
- Hue parameter range	(-0.05, 0.05)
- Saturation range	(0.95, 1.05)
- Motion blur	Motion in x-direction (horizontal)

Supplementary Method S2. Calculation of dissimilarity between frames

Since each video (30 frames per second) was composed of many similar frames, using all frames to train the AI system may not be beneficial. We selected only significantly different frames to train the AI system, by calculating dissimilarity between frames, and used only frames with dissimilarity values greater than a threshold for training. We calculated Mean Absolute Difference (MAD) to represent dissimilarity between frames using the following formula:

$$MAD_{frame\ i\ vs\ j} = \frac{1}{N} \sum_{xy \in S} \left| I_{xy,i} - I_{xy,j} \right|$$

where i and j are the indices of frames being compared.

N is the number of pixels in the frame.

xy is the (x, y) coordinate in the frame.

S is the set of all possible coordinates in the frame.

$I_{xy,i}$ is the normalized intensity value (scale from 0 to 1) at coordinate xy of frame i.

We iteratively sampled frames with significant differences. A pair of frames are considered significantly different if $MAD > 0.1$.

Supplementary Method S3. Evaluation metrics for the AI system

We evaluated the AI performance by the following metrics:

Per-lesion detection rate. This was the primary outcome in our study. Detection results in all frames of each lesion were aggregated into a per-lesion detection result. Previous reports on the AI system for detection of polyps in colonoscopy described criteria for aggregating per-frame detection into per-polyp detection result by considering a polyp as detected only if the proportion of frames with detected polyp reached a certain cut-off.[1,2] We used the same approach with additional consideration on the difference in characteristics between polyps in colonoscopy and FLLs in ultrasonography. Polyps appear during colonoscopy as objects with depth information in colonoscopy videos. Their appearance remains relatively similar in every frame. In contrast, FLLs seen in ultrasound are a '2-dimensional slice' through the lesion. When the slice passes through the center of FLL, the corresponding frame shows the lesion having a size very similar to its real diameter. When the slice passes through the peripheral region of an FLL, the lesion may appear very small and faint. Because our full-length videos consisted of only slices of FLLs, we considered an FLL as detected if the AI system detected it in at least 1% of the frames containing FLL.

False positive detection rate. When the AI system falsely detected other organ structures as FLLs, these frames were counted as false positive detections. We calculated the false positive detection rate by dividing the number of frames with false positive detection by the total number of frames in each video. We reported the median false positive detection rate, calculated across all videos in the test set.

Supplementary Table S1. Characteristics of focal liver lesions in the test set

	HCC	Cyst	Hemangioma	FFS	FFI
N	23	34	27	30	13
**Size**					
< 1 cm	1 (4.3%)	9 (26.5%)	5 (18.5%)	8 (26.7%)	0
1 to < 2 cm	14 (60.9%)	23 (67.6%)	16 (59.3%)	20 (66.7%)	9 (69.2%)
2 to < 3 cm	5 (21.7%)	1 (2.9%)	5 (18.5%)	2 (6.7%)	4 (30.8%)
≥ 3 cm	3 (13.0%)	1 (2.9%)	1 (3.7%)	0	0
**Echogenicity**					
Hypoechoic	7 (30.4%)	0	5 (18.5%)	30 (100%)	0
Hyperechoic	8 (34.8%)	0	21 (77.8%)	0	13 (100%)
Heterogeneous	8 (34.8%)	0	1 (3.7%)	0	0
Anechoic	0	34 (100%)	0	0	0

FFS, focal fatty sparing; FFI, focal fatty infiltration

Supplementary Figure S1. Example lesions in the test set not detected by the final AI system (*Model C*). Labels are shown as blue bounding boxes in right panels. Figure 1a shows a small faint hyperechoic hemangioma located in the subcapsular area of liver. Figure 1b shows an atypical faint hypoechoic hemangioma in the background of steatotic liver parenchyma. Figure 1c shows a small cyst indistinctively appeared at the periphery of the frame when the ultrasound probe was moving and was not focusing on the lesion.

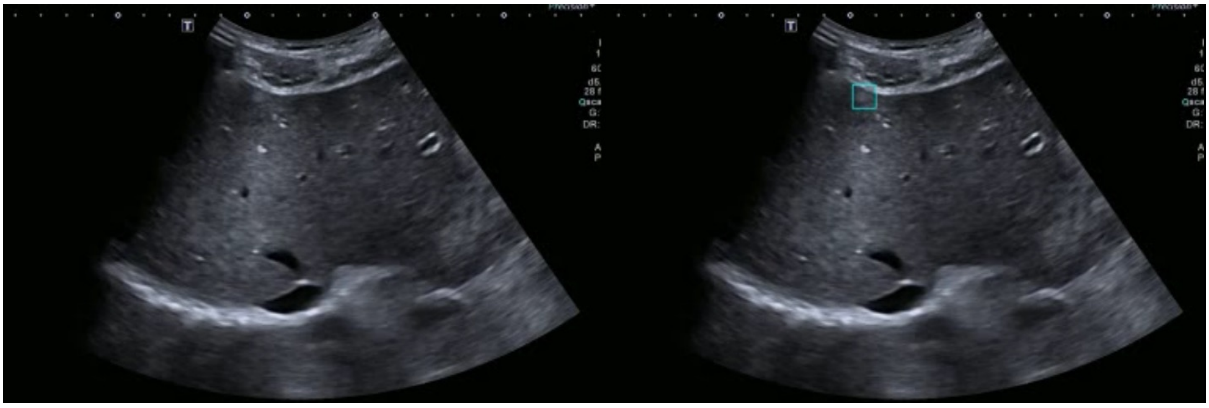

1a

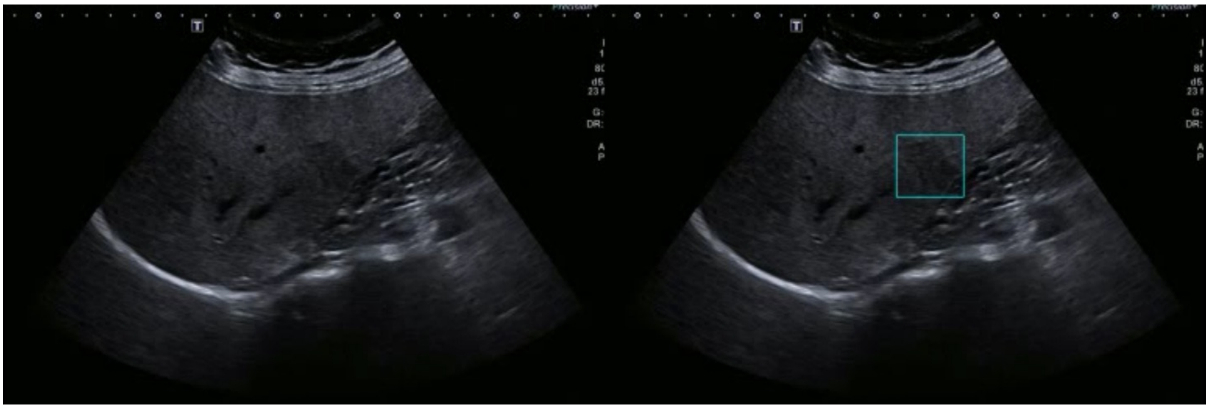

1b

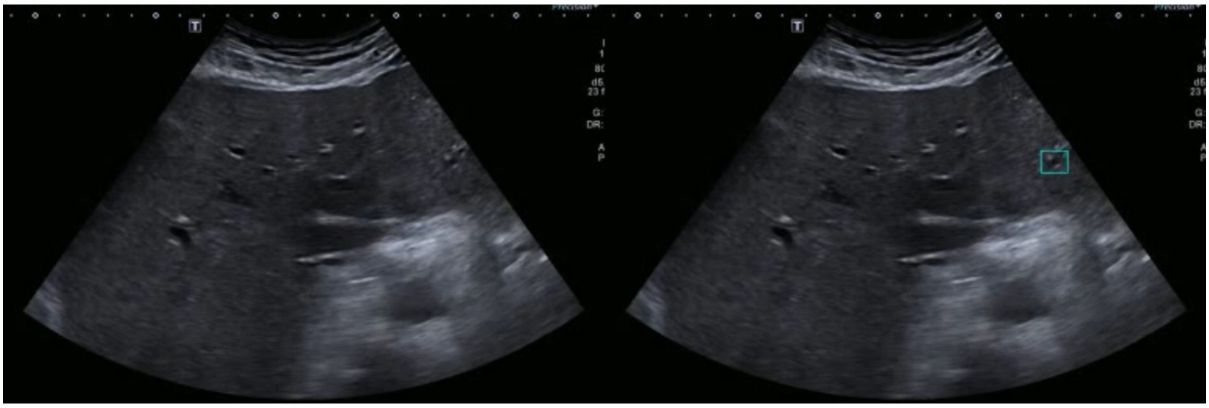

1c

Supplementary Video S1

Supplementary Video S1 shows our AI system detecting a focal liver lesion in an ultrasound video. The top left panel shows original video. The top right panel shows video with labeled ground truth location of the lesion. Bottom left panel shows prediction of the AI system before applying heuristic method. Bottom right panel shows prediction of our final AI system (*Model C*) after applying heuristic method. The heuristic method allows predicting detection only if the AI system predicts at least 2 consecutive frames as having a lesion.

References

1 Misawa, M. *et al.* Artificial Intelligence-Assisted Polyp Detection for Colonoscopy: Initial Experience. *Gastroenterology* **154**, 2027-2029.e2023, doi:10.1053/j.gastro.2018.04.003 (2018).

2 Urban, G. *et al.* Deep Learning Localizes and Identifies Polyps in Real Time With 96% Accuracy in Screening Colonoscopy. *Gastroenterology* **155**, 1069-1078.e1068, doi:10.1053/j.gastro.2018.06.037 (2018).
